# Woman’s Needs and Satisfaction Regarding the Communication with Doctors and Midwives during Labour, Delivery and Early Postpartum

**DOI:** 10.3390/healthcare9040382

**Published:** 2021-04-01

**Authors:** Barbara Baranowska, Paulina Pawlicka, Iwona Kiersnowska, Alicja Misztal, Anna Kajdy, Dorota Sys, Antonina Doroszewska

**Affiliations:** 1 Department of Midwifery, Centre of Postgraduate Medical Education, 01-813 Warsaw, Poland; barbara.baranowska@cmkp.edu.pl; 2 Institute of Psychology, University of Gdansk, 80-309 Gdańsk, Poland; 3 Department of Obstetrics and Perinatology, Medical University of Warsaw, 02-091 Warsaw, Poland; ikiersnowska@gmail.com; 4 St. Sophia’s Specialist Hospital, 01-004 Warsaw, Poland; alicja.misztal@gmail.com; 5 Department of Reproductive Health, Centre of Postgraduate Medical Education, 01-813 Warsaw, Poland; akajdy@cmkp.edu.pl (A.K.); dsys@cmkp.edu.pl (D.S.); 6 Department of Medical Communication, Medical University of Warsaw, 02-091 Warsaw, Poland; skm@wum.edu.pl

**Keywords:** communication, childbirth, satisfaction, quality of care

## Abstract

The study aimed to identify the difference in communication needs of women giving birth and women during early postpartum. An additional goal includes the analysis of the experience and communication needs through the context of a woman’s approach to childbirth. The study is a cross-sectional, self-report survey; 521 women between 5 and 10 days after birth participated in the study. Women perceived information provided by the medical staff as the most helpful aspect of verbal communication both during labour and early postpartum. Maintaining eye contact with the medical staff was perceived as the most helpful aspect of non-verbal communication. Women were more satisfied with communication during labour and birth than in the maternity ward and those after non-instrumental childbirth were more satisfied with communication compared to the instrumental birth group. Women perceiving childbirth as the natural, physiological process considered verbal and non-verbal communication during and after childbirth as less helpful than women perceiving birth as more risky and requiring interventions. The results of the study emphasize the importance of verbal and non-verbal communication during birth and early postpartum and at the same time different communication needs during these two time points. It also showed that women who perceive labour as a physiological process seem to be less dependent on the communication with the medical staff than women who accept medical interventions during labour and birth as necessary.

## 1. Introduction

Communication constitutes one of the eight domains of the quality of the perinatal care within the framework proposed by the World Health Organization [1]. The rapport between the woman and the midwife is largely built through communication [2,3]. Communication may become the element strengthening the feeling of safety and self-efficacy of the birthing woman, which is vital during labour and significantly affects the woman’s satisfaction with the perinatal care [2]. According to Nunes et al., “effective communication by maternity care staff can help a woman during labor and birth to have a positive birth experience” [4]. With regard to the perinatal care it should be ensured that “communication with women and their families is effective and responds to their needs and preferences” [5]. At the same time, no agreement has been reached with regard to the definition of effective communication in the perinatal care [6,7]. The qualitative research conducted by the Downe team describes the needs of women in labour and their expectations towards maternity and perinatal care, which to a large degree concerned communication with staff [8]. The research results show that providing the information in a clear manner and the staff’s interest in women’s needs and fears are of key importance for these women. This is primarily reflected in the staff being able to listen to what the woman says during labour [9].

The studies dedicated to communication between the maternity care staff and women are mainly aimed at the effectiveness of perfecting the communication skills [6,10]. Secondly, they aim at lowering the barriers to the facility-based delivery in low- and middle-income countries [11,12]. They most commonly are therefore presented in the context of providing patients with respectful care [13,14]. The most investigated communication skills include active listening, giving information, and obtaining an informed consent [7].

Non-verbal communication is one of the key communication skills in the midwife’s work. According to The Nursing and Midwifery Council’s Code, the midwife should “use a range of verbal and non-verbal communication methods, and consider cultural sensitivities, to better understand and respond to people’s personal and health needs” [15]. The research on communication skills of midwives indicates a relationship between women’s satisfaction and with midwives’ both verbal and non-verbal communication skills [16]. 

During natural childbirth, the woman’s body partially withdraws from cognitive processes. An instinctive action related to the secretion of hormones dominates her behaviour, thus non-verbal communication may be more attuned to the process of birth itself. A conversation and expecting rational responses from the woman in labour may disturb the natural flow of childbirth [17]. In the postpartum period, to a large degree the woman’s attention is focused on the child and the search for information and assistance in properly taking care of the baby, hence underlining the importance of verbal communication. This different nature of tasks and activities during childbirth and early postpartum may be associated with different needs for verbal and non-verbal methods of communication with the medical staff. In case of physiological birth, without medical interventions and in conditions that support the natural secretion of oxytocin, cognitive functions are minimized to allow activation of the allocortex crucial for hormone secretion and instinctive behaviours [17,18]. In case of instrumental birth or caesarean section (particularly elective), minimizing cognitive functions may be more difficult and this may lead to different communication needs among these women [17]. Women’s experience contributing to the final assessment of satisfaction with care received at the hospital may be divided into the experience before labour (usually at the admission), during labour (in the labour room) and after labour (in the maternity ward). The results of previous studies point to differences in the women’s feelings related to their stay at the given hospital unit [19]. 

The aim of the present study was to verify if there are differences in the needs of the women in labour and early postpartum related to the verbal and non-verbal communication with the medical staff. Secondly, we wanted to assess if these needs are differentiated by the perception of the risk of childbirth for the mother and the baby. Thirdly, we wanted to analyse the possible differences in the communication needs with regards to the instrumentalization of delivery.

## 2. Materials and Methods

In this cross-sectional, descriptive study, a survey was administered to 521 women from two hospitals, A and B, in Warsaw, Poland. Facility A (tertiary clinical hospital, 4 labour and delivery rooms, the annual total number of births 2300, caesarean section rate 34%). Facility B (tertiary clinical hospital, 10 labour and delivery rooms, the annual total number of births 6500, caesarean section rate 30%; in-hospital midwifery-led birth centre with the annual number of births between 600 and 800 where only women in physiological pregnancy are admitted, only non-pharmacological pain relief is available and medical interventions are kept to a minimum) [20]. Both facilities were rated very high on the hospital ranking organized by the non-governmental organization active in the area of maternity health in Poland. These ratings may suggest that the medical personnel in both hospitals presents high communication skills.

The survey was distributed to 550 women; 523 surveys were returned, out of which 2 surveys were lacking sociodemographic information. The recruitment rate was 95%. Participants filled in the paper-and-pencil self-administered questionnaire directly before the discharge from the hospital (5–10 days after labour). The survey was conducted in Polish. Content validation for the questions concerning the communication needs and the one regarding perception of childbirth was performed by the expert panel including three midwives, two psychologists and one obstetrician. In the validation process, the questionnaire received the following value Scale-level Conent Validity Index (S-CVI) = 0.80. The coefficients of reliability were Cronbach’s alpha = 0.71. The study was anonymous, and participation was voluntary. All participants expressed their consent to take part in the study.

### 2.1. Communication Needs and Satisfaction with Communication during Birth and Early Postpartum

The survey included questions about women’s needs for verbal and non-verbal communication with the medical staff during childbirth and in the maternity ward. Four questions related to this aspect with regards to the experience during birth and the same questions were asked with regards to the postpartum experience. The instruction was as follows: “Please imagine or recall the birthing experience and indicate whether during birth/Please imagine or recall the postpartum experience and indicate whether in the maternity ward: (1) Asking a large number of questions by doctors/midwives; (2) Maintaining eye contact with the midwife; (3) Receiving a large amount of information from the doctor/midwife; (4) Communicating with the midwife by touch”. Then participants were asked to mark one of three possibilities of answer to each question: disturbed me; had no impact; helped me. Asking questions and providing information were assigned to the verbal communication while touching and maintaining eye contact were assigned to the non-verbal communication.

A value representing the level of communication helpfulness with the value 1 representing “it disturbed me”, 2—“did not matter” and 3—“it was helpful” was assigned to each response.

Two additional questions regarding the satisfaction from the received care during birth (“Please, mark your level of satisfaction with the communication with the medical staff during your stay in the hospital during labour and birth”) and early postpartum (“Please, mark your level of satisfaction with the communication with the medical staff during your stay in the hospital maternity ward”) were included in the survey. Participants responded on a 5-point Likert scale from 1 (definitely not satisfied) to 5 (definitely satisfied).

The following hypotheses were formulated: H1: Non-verbal communication is more helpful during labour and birth than verbal communication. H2: Verbal communication is more helpful during early postpartum than non-verbal communication. H3: Non-verbal communication is more helpful for women during vaginal birth than verbal communication. H4: Verbal communication is more helpful for women during instrumental birth than non-verbal communication.

To compare women’s communication needs and their level of satisfaction with communication during labour and birth and their subsequent hospital stay in the maternity ward, dependent sample *t*-test was used, with the timepoint regarding childbirth (during childbirth vs. during early postpartum) as the independent variable and particular aspects of communication and the level of satisfaction as dependent variables.

To assess the differences in women’s communication needs between vaginal births and instrumental births, independent sample *t*-test was used with the type of birth as the independent variable (vaginal birth vs. instrumental birth) and particular aspects of communication and the level of satisfaction from communication as dependent variables.

### 2.2. Perception of the Childbirth Risk

Perception of the childbirth risk was assessed with one question: “Please, mark one answer that best reflects your opinion about childbirth” with 3 possibilities provided: (1) Woman is capable of giving birth to a child and usually no medical intervention is needed; (2) Childbirth is a normal physiological process, but medical interventions are necessary to carry out childbirth and give birth to a healthy child; (3) Childbirth is always at high risk for the baby and the mother. Study participants were divided into three groups according to the marked option: women who perceive childbirth as an instinctive process (the physiology group), women who believe that medical interventions are necessary for the birth of a healthy child (the intervention acceptance group) and women for whom childbirth is a high-risk event not only for the mother but also for the child (the high-risk group). 

The following hypotheses were formulated: H5: For women who believe that a woman is capable of giving birth to a child and usually no medical interventions are needed, non-verbal communication is more helpful during labour and birth than for women perceiving childbirth as risky for the baby and the mother (the intervention acceptance group and the high-risk group). H6: For women perceiving childbirth as the risky process (the intervention acceptance group and the high-risk group) verbal communication is more helpful during labour and birth than for women from the physiology group. 

To assess the differences between the perceived risk of childbirth and the particular aspects of communication, a one-way ANOVA test was used with the perceived level of risk as an independent variable and the level of helpfulness of verbal and non-verbal communication and the level of satisfaction with communication during childbirth and during early postpartum as dependent variables.

### 2.3. Other Descriptive Variables

The questions about demographic characteristics were included in the survey (age, education level, marital status, parity) and the factors which could significantly impact the feelings of connection with labour and birth: mode of delivery, epidural use, presence of a birth partner, place of birth (birth centre or standard delivery ward), interventions—labour induction or augmentation. We have differentiated the group of women who experienced an instrumental delivery (caesarean section or vaginal birth with use of vacuum or forceps) and the group of women after the vaginal delivery (vaginal birth with or without medications). 

### 2.4. Statistical Analysis

The data obtained from the study were analysed using the SPSS 26.0 (IBM, Armonk, NY, USA) package software. Frequency, percentage, and Pearson correlation coefficient were calculated. In addition, student dependent and independent *t*-test and the analysis of variance (one-way ANOVA) with post hoc Tukey test were performed in order to validate the research questions. 

The study received the approval of the Ethics Committee at the Medical University of Warsaw, ref. No. AKBE/232/2017.

## 3. Results

### 3.1. Characteristics of the Study Group

A total of 521 women (75.3% aged between 26 and 35 years; 85.5% with higher education) participated in the study. Of these, 96.5% gave birth in the maternity wards in public hospitals in Warsaw, Poland and 3.5% (18 women) in the birth centre. Note that 52% were primiparas; 69% had vaginal birth (16.2% had unmedicated vaginal birth and 53.2% medicated with epidural, Entonox and/or oxytocin induction). In addition, 14.3% had planned and 16.4% unplanned c-section, and 72% of women had a close person accompanying them during labour and/or birth. 

### 3.2. Communication Needs during Childbirth/Early Postpartum and Satisfaction with Communication

Both verbal (providing information, asking questions) and non-verbal (touching, maintaining eye contact) communication with the medical staff was perceived as helpful during childbirth and early postpartum. However, women differently assessed the same aspects of communication in these two situations (Table 1). Verbal communication was more often assessed as helpful in the maternity ward than during labour and birth (thus the hypothesis 2 has been confirmed). The same pertains to maintaining the eye contact with the midwife. However, being touched was more often marked as helpful during labour and birth than in the maternity ward (thus partially confirming the hypothesis 1). Within the verbal communication, providing information was more often perceived as helpful than asking questions, and within the non-verbal communication maintaining the eye contact was more helpful than being touched (Table 1).

General satisfaction with communication with the medical staff was high both during labour: 4.74 (*SD* = 0.565; scale 1–5) and early postpartum: 4.58 (*SD* = 0.665; scale 1–5) (Table 1). However, women were more satisfied with communication during labour and birth than in the maternity ward (early postpartum).

The communication needs and satisfaction were also analysed with respect to the mode of delivery. The results of the independent *t*-test analysis showed that the only aspect of communication that differentiated the groups was related to asking questions in the maternity ward (early postpartum), perceived as more helpful by the women after vaginal birth (Table 2). Significant differences were however found with regards to the satisfaction with communication during labour/delivery and early postpartum. Women after the vaginal delivery were more satisfied with the communication with the medical staff in both situations. Thus hypotheses 3 and 4 were not confirmed.

### 3.3. Perception of Childbirth Risk and Communication Needs

Finally, we wanted to verify whether perception of the childbirth risk differentiates the communication needs and satisfaction of the participants during childbirth and early postpartum. Of the participating women, 15.8% indicated that in their opinion birth is the physiological process and no interventions are needed; 64.6% of participants perceived birth as a normal physiological process, but medical interventions are in their opinion necessary to give birth to a healthy child; 18.2% of women perceived childbirth as a highly risky process for the baby and the mother.

One-way ANOVA was conducted with the perception of childbirth risk as the differentiating variable and on each of the communication aspects as dependent variables (Table 3). 

The results of the analysis showed the main effect of perception of childbirth risk for both aspects of verbal communication (the Tukey post hoc test indicated that women convinced that labour and birth constitute the physiological process perceived questions asked during labour and in the maternity ward and information provided in the maternity ward as more disturbing than the two other groups and information from the medical staff during labour as more disturbing than the group perceiving birth as the highly risky experience), thus confirming hypothesis 6. With regards to the non-verbal communication needs, the main effect was found for touch during childbirth and early postpartum and for the eye contact postpartum (similarly to the verbal communication needs, according to the results of the post hoc Tukey test women perceiving childbirth as the natural, physiological process considered being touched during and after childbirth as less helpful than the two other groups, and maintaining eye contact with midwives in the maternity room as less helpful than the women perceiving birth as a highly risky process), thus hypothesis 5 has not been confirmed. Perception of childbirth risk did not differentiate the groups when satisfaction with communication with the medical staff both during and after childbirth were analysed.

## 4. Discussion

Our study filled the gap in the knowledge, as identified by the Guideline Development Group (GDG), related to the communication characteristics in the area of perinatal care, which may influence the labour experience [19]. Satisfaction with the communication with the medical staff is important and it constitutes a significant factor influencing the general satisfaction with birth care and can improve women’s experience of childbirth [21,22]. Lower satisfaction with the communication with the staff may result in lowering of postpartum mood observed during women’s stay in the maternity ward. Fatigue, tension, and anxiety may negatively influence the assessment of the staff. Women experience a range of emotional and hormonal changes in the early perinatal period, which results in often expecting the additional support expressed via communication [23]. 

Our study indicates a very high level of satisfaction with the communication with the staff both during labour and in the maternity ward. The results are in concordance with the data obtained by Adamska et al. from a representative group of Polish women, which concluded that most of the women in labour thought that the staff communicated with them in an appropriate manner during their hospital stay [19]. Similar results were presented in the group of women in the perinatal care in Iran, and in Egypt, where a vast majority were satisfied with respect to the communication with the staff [24,25]. Simultaneously, the experience connected with childbirth is at times more positive in the early period after labour when compared to the one subsequently reported. Research in Denmark demonstrated that the measure of satisfaction with labour assessed directly after labour may be distorted by relief at finishing a period of tenseness and uncertainty accompanying the pregnancy and happiness with giving birth to a healthy child [26]. Thus, this fact should be taken into account when assessing the study results. 

Women indicated receiving information from the staff as the most desired aspect of verbal communication. This applied to communication both during labour and in the maternity ward. Information obtained from the persons looking after the woman increase her ability to take part in the decision making and, by the same token, increase her external control. The increased feeling of external control positively influences the labour experience [27] and allows to make decisions about their childbirth [28]. Women in labour need informational support provided by the staff. “Offering the woman and her family the information they need in a clear and concise manner” is one of the recommendations regarding effective communication between maternity care providers and women in labour [29]. Feeling informed (about birth process, personnel roles, child’s health during birth and about how to feed the child) significantly affects birth experience [30]. In our study, verbal communication was more often assessed as helpful in the maternity ward than during labour and birth. It may result from the fact that the woman in early postpartum is focused on receiving messages and information about the baby, so she may prefer the verbal communication, focusing on the newborn, as well as touching and holding him/her. Breastfeeding support and information are also important aspects at this point, affecting both overall birth experience [30] as well as breastfeeding duration [31].

Touching and keeping an eye contact by the medical staff was also perceived as helpful both during labour and early postpartum. However, touch was perceived as more helpful in labour, while maintaining the eye contact was more often indicated as helpful in the maternity ward. The touch was considered an aspect of non-verbal communication [32]. Touch was the most commonly used form of contact by midwives participating in the study in Brasil and Cape Verde [33]. The touch during an interaction with the physician affects the positive assessment of the carer’s empathy [34]. The touch in labour is also considered as a way of coping with intense pain [35]. Despite the fact that for most women in labour touch was helpful because it aided them in coping with the experience, the research shows that the touch is not desired in every labour stage and not every woman perceives it as something comfortable [36]. It is also important to acknowledge that there are different types of touch, including expressing touching and therapeutic touching that play an important role in care during labour [37,38]. Women may also have very different needs when it comes to types of touch during labour. Thus, seeking consent from women is very important. In many situations, a supportive non-verbal communication instead of directive verbal communication, offered in accordance with the rule “the less we do, the more we give”, may be more beneficial for the labour progress, woman’s satisfaction, as well as for the midwives themselves [39].

The lower satisfaction with communication in the maternity ward (early postpartum) than with communication during labour observed in our study may be connected with the change in the system of care after childbirth. In accordance with the national guidelines, the woman in labour has the continuity of care ensured and is taken care of by a single midwife, dedicated to that woman. However, in the maternity ward, one midwife looks after a few or a dozen women and their responsibilities are often related to particular activities (vaccinating, giving bath to children, lactation assistance) rather than looking after a particular woman. Such mode of work influences the time limits during which the staff responds to women’s needs, decreases the chance of establishing a rapport with the woman, and may hinder communication. The results of other studies indicate that women tend to assess communication in a more positive way when a smaller number of persons look after them, which in turn gives them a bigger chance to build a rapport and engagement in making decisions regarding the care [7].

The lower satisfaction with communication between women and the staff was observed also in women experiencing the instrumental birth (caesarean section or vaginal with vacuum) compared to women after the vaginal one. This may result from the stress experienced by the woman herself, the medical staff focused on efficient performance of the operation or/and lack of time to provide information to a birthing woman. Results of other studies also indicate that particularly women after emergency caesarean sections report lower satisfaction with labour and care during labour [40].

Finally, the results of our study emphasize the role of personal attitude towards childbirth in communication needs of birthing women. The majority of the participating women stated that medical interventions are necessary to bear a child. The smallest group considered labour and birth as a physiological process that does not need medical interventions. However, for these women, intensive communication during labour and birth (asking many questions, providing large amounts of information and being touched) and in the maternity ward was more disturbing and less helpful than for both other groups. These results may underline their need to experience the birthing process on their own, at their own pace. During labour, the neo-cortex (rational brain) needs to be muted in order to activate the limbic system taking part in an effective secreting of hormones [17]. A conversation and expecting rational responses from the woman in labour may disturb the natural flow of childbirth. Such “immersion” in labour is observed during natural labour [17]. Results of other Polish studies indicate that women childbearing at home (homebirth is usually the natural, physiological birth) report a high level of self-efficacy, higher control over pain and more frequent reinterpreting the experience of pain comparing to women giving birth at a hospital indicating their ability and will to be the guides of their birthing process [41]. Yet, as our study showed, in a medicalized, instrumental, or operational labour, the cognitive system is stimulated, so asking questions was perceived as less disturbing for these women.

One of the limitations of the study is the lack of information regarding the initial preferences of women on the types of communication. Also, we did not analyse whether women had any communication problems unrelated to labour and birth, such as ASD (Autism Spectrum Disorder), increased sensitivity to touch, past experience of sexual violence. The presence of such problems among the participants could have had an impact on the results. In addition, the study analysed women’s satisfaction with communications with medical personnel without separating between midwives and doctors and their communication skills. In the future, it would be interesting to see whether there are differences in how women perceive the helpfulness of verbal and non-verbal communication separately for both these professions. Another limitation of the study is the lack of open-ended questions allowing women to comment and express opinions in case none of the provided answers reflected their experience. Finally, information on income level and social support were not collected.

## 5. Conclusions

The findings of the study underline the importance of the attitude towards childbirth for the communication needs. Women who perceived labour as a physiological process seemed to be less dependent on the communication with the medical staff than women who accepted medical interventions during labour and birth as necessary. It is therefore meaningful to gather information from women about their preferences regarding the means and intensity of communication during childbirth and early postpartum and become attentive to changes in their needs according to the context and situation. It is also important to remember the importance of communication with birthing women during the instrumental delivery, as the results point to the significantly lower satisfaction with care in these situations. Training sessions should be organized for persons providing care for women in labour and during early postpartum and their babies in order to raise awareness about women’s communication needs during this sensitive and important moment of life.

## Figures and Tables

**Table 1:** Descriptive statistics and results of the dependent *t*-test analysis of communication needs and communication satisfaction during childbirth and early postpartum.

Communication Aspect	*N*	*M*	*SD*	*t*	*df*	*p*
questions—birth	518	2.18	0.766	−9.245	517	0.000
questions—postpartum	518	2.50	0.733			
information—birth	516	2.79	0.502	−4.792	515	0.000
information—postpartum	516	2.88	0.361			
eye contact—birth	515	2.72	0.490	−3.712	514	0.000
eye contact—postpartum	515	2.80	0.432			
touching—birth	515	2.64	0.547	3.751	514	0.000
touching—postpartum	515	2.55	0.553			
satisfaction—birth	517	4.74	0.565	5.216	516	0.000
satisfaction—postpartum	517	4.58	0.665			

**Table 2:** Descriptive statistics and results of the independent *t*-test analysis of communication needs and communication satisfaction during and after instrumental and non-instrumental delivery.

	Non-Instrumental Birth			Instrumental Birth					
Communication Aspect	*N*	*M*	*SD*	*N*	*M*	*SD*	*t*	*df*	*p*
questions birth	336	2.18	0.754	182	2.18	0.790	0.045	516	0.964
questions postp.	337	2.55	0.693	184	2.42	0.792	1.907	519	0.057
information birth	335	2.80	0.478	183	2.78	0.544	0.456	516	0.648
information postp.	336	2.88	0.356	183	2.89	0.368	−0.040	517	0.968
eye contact birth	335	2.71	0.492	180	2.74	0.486	−0.751	513	0.453
eye contact postp.	337	2.81	0.403	184	2.79	0.483	0.481	519	0.631
touching birth	337	2.63	0.553	179	2.68	0.554	1.096	514	0.273
touching postp.	336	2.54	0.545	183	2.58	0.567	−0.740	517	0.460
Satisfaction—birth	336	4.84	0.409	182	4.57	0.745	5.225	516	0.000
satisfaction postp.	336	4.65	0.618	183	4.43	0.722	3.698	517	0.000

**Table 3:** Results of the one-way ANOVA analysis with the perception of childbirth risk as the differentiating variable and each of the communication aspects as dependent variables.

Communication Aspect	*F*	*df*	*p*	*p*η2
questions birth	11.932	2	0.000	6.712
questions postp.	8.991	2	0.000	4.686
information birth	2.902	2	0.056	0.712
information postp.	6.909	2	0.001	0.870
eye contact birth	1.811	2	0.165	0.434
eye contact postp.	4.479	2	0.012	0.826
touching birth	5.078	2	0.007	1.497
touching postp.	8.703	2	0.000	2.582
satisfaction—birth	0.259	2	0.772	0.083
satisfaction postp.	0.836	2	0.434	0.371

## Data Availability

The data presented in this study are available on request from the corresponding author.
